# Experimentally paired high- and low-resolution confocal fluorescence microscopy dataset for deep-learning super-resolution imaging of tooth dentin porosity

**DOI:** 10.1016/j.dib.2026.112666

**Published:** 2026-03-10

**Authors:** Lauren Anderson, Seunghwan Goldmund Lee, Kathryn Grandfield, David Rousseau, Aurélien Gourrier

**Affiliations:** a Univ. Grenoble Alpes, CNRS, LIPhy, Grenoble F-38000, France; b School of Biomedical Engineering, McMaster University, 1280 Main St. W, Hamilton, ON L8S 4L7, Canada; c Pusan National University, Department of Opto & Cogno Mechatronics Engineering, 57, Oryundae-ro, Geumjeong-gu, Busan 4624, Republic of Korea; d Department of Materials Science and Engineering, McMaster University, 1280 Main St. W, Hamilton, ON L8S 4L7, Canada; e Laboratoire Angevin de Recherche en Ingénierie des Systèmes (LARIS), UMR INRAe-IRHS, Université d’Angers, 62 Avenue Notre Dame du Lac, 49000 Angers, France

**Keywords:** Dentinal tubule network, Mechanosensing, Fast-scanning, Biology-driven machine learning

## Abstract

The proposed dataset provides experimentally acquired high- and low-resolution confocal laser scanning microscopy images of dentin porosity, split into classified image patches that can be used for paired or unpaired super-resolution training. Porous dentinal tubule and branch channels represent a vast network and can be used as a model for the mechanosensory system of teeth. The large quantity and small size of tubules and branches present an imaging challenge, requiring high resolution images at a large field of view in order to image statistically relevant regions of the tooth and fully model the porosity network. Imaging more quickly increases the potential of the field of view that can be acquired, however in return decreases resolution. Deep-learning based super-resolution could be used on low-resolution images to restore high resolution information from the image. A fluorescently stained tooth slice was first imaged with 100×100 nm^2^ pixel size to acquire data at the Nyquist resolution based on the theoretical point spread function of the microscope (216 nm), and low-resolutions images of the same field of view were acquired at 200×200 nm^2^, 400×400 nm^2^, and 800×800 nm^2^ pixel sizes without changing the microscope objective to ensure image region matching. A total of six regions were imaged at 5.1 µm in depth for a total of 18 slices per region. Acquired images were registered to ensure data were paired, and images were further patched and classified as one of 3 classes based on the type and scale of porosity present in each patch. This provides a unique database of classified image patches that can be used for paired or unpaired super-resolution training, providing real examples in all resolutions.

Specifications Table

**Table:** 

Subject	Biology, Computer Sciences
Specific subject area	Confocal fluorescent microscopy of dentin microscopic porosity network for super-resolution deep learning imaging.
Type of data	Raw, Analysed.
Data collection	2D images of dentin porosity were acquired using a Leica SP8 (Leica Microsystems CMS GmbH, Wetzlar, Germany) confocal laser scanning microscope with a 40x oil objective of 1.3NA. A polished slice sample of 200 µm in thickness was cut from a 28-year-old female third-molar and stained with 0.02 %w with RhB in Glycerol. The sample was mounted between 2 cover-slides sealed with glue. 3D confocal stacks of 18 images were acquired in six regions at 100×100 nm^2^, 200×200 nm^2^, 400×400 nm^2^, and 800×800 nm^2^ pixel sizes. Images were saved individually as 8-bit grayscale .tiff files. Low resolution stacks were registered to the high-resolution stack using ANTSPY version in 0.4.2 in python version 3.10, which uses mutual information to find the rigid transformation of the images. Image patches of 128×128 pixels were extracted and classified in 3 groups of features based on the type of biological structure in each patch containing branches, tubules or both. All resulting high and low-resolution patches for each class were finally saved as 8-bit grayscale .tiff files for each resolution.
Data source location	Institution: Laboratoire Interdisciplinaire de Physique (LIPhy), UMR 5588 CNRS Université Grenoble Alpes. City/Town/Region: Saint-Martin-d’Hères Country: France Latitude/longitude: 45.193335, 5.763294
Data accessibility	Repository name: Multi-resolution confocal fluorescence microscopy of human dentin porosity Data identification number: https://doi.org/10.5281/zenodo.17185790 Direct URL to data: https://zenodo.org/records/17185791 The Python code used to analyze the data and produce the results is available without restriction under the BSD-3 licence conditions, on the following Gitlab repository of the University Grenoble Alpes: https://gricad-gitlab.univ-grenoble-alpes.fr/mintissliphy/studentscodes/pysrmintiss.git (commit 36cba3ca04c6a7fd72062f05a4b925314bbdb0ee)
Related research article	L. Anderson, L. Chatelain, N. Tremblay, K. Grandfield, D. Rousseau, and A. Gourrier, “Biology-driven assessment of deep learning super-resolution imaging of the porosity network in dentin,” arXiv:2510.08407, doi: 10.48550/arXiv.2510.08407

## 1 Value of the Data

• This data set consists of unique experimentally acquired pairs of high- and low- resolution confocal fluorescent images of microscopic dentin porosity registered at the pixel level. The 2D images typically represent slices of a 3D network of tubular porosity with two characteristic diameters.
• The data are primarily intended for training and testing deep learning super-resolution models with ground truths for generated image assessment.
• To allow a balanced training of super-resolution machine-learning models with even representations of the two distinct scales of biological porosity, image pairs were further sorted in three corresponding groups: one for each scale and a third for mixed features that could not be separated. Particular care was taken to ensure a fair representation of the smallest porosity branches which are critical connectivity components of the network, for accurate biological modelling.
• Our data could also be valuable for other deep-learning generative imaging purposes, including testing existing or new Image Quality Assessment metrics that aim to quantify generated image fidelity to a corresponding ground truth (e.g. full reference methods).

A full description of possible use cases supporting the above claims can be found in Anderson et al. [1].

## 2 Background

The dentinal porosity network is assumed to play a key role in the mechano-sensory function of the tooth [2,3]. Visualizing the microscopic diameter of tubules and branches (1–3 µm and 300–700 nm diameter, respectively [4]) requires high-resolution imaging, which inevitably comes at the cost of limited field of view (FOV). Fluorescent confocal microscopy provides a good imaging method for visualization of the porosity network [5]. Fast acquisition of low-resolution images followed by image restoration could bridge the gap between fast-scanning and high-resolution imaging. Deep learning super-resolution imaging currently outperforms traditional restoration methods [6]. However, many generative models require paired datasets at the pixel level which can be incredibly challenging to acquire experimentally. As a workaround, synthetic low- or high-resolution images are frequently used to train deep-learning models. However, models trained with synthetic data always shows a domain shift with real world data which comes with decreased performance when applied to real images [7]. Unpaired models aim to overcome the need for experimentally paired data which are often difficult to obtain in practice. However, the lack of ground truth to assess the quality of generated images severely complicates the quantification of model performance. The acquisition of this dataset was thus motivated by the desire to perform deep learning super-resolution of dentin porosity, the need for experimentally paired data for optimal model training and the importance of ground truth data for proper quantification of both supervised or unsupervised model performance.

## 3 Data Description

The main repository contains one main folder called ‘MultiRes_ConfocFluo_HumanDentin’ which is then separated into 3 folders: “RawImages”, “RegisteredImages” and “ImagePatches128”. Full folder hierarchy including subfolders and file names is described below and summarized in Table 1.

**Table 1:** Folder contents and file names for all data folders in the repository.

Dataset	Main Folder	Subfolders			Filenames
MultiRes_ConfocFluo_HumanDentin	RawImages	Sample01	ROI1 ROI2 ROI3 ROI4 ROI5 ROI6	HR x2 x4 x8	s01_roiX_z0000.tif… s01_roiX_z0017.tif
	RegsiteredImages		ROI1 ROI2 ROI3 ROI4 ROI5 ROI6	x2 x4 x8	s01_roiX_z0000_reg.tif… s01_roiX_z0018_reg.tif
	Image Patches128	Classified	both branches tubules	HR x2 x4 x8	img_00000_s01_roiX.tiff… img_N_s01_roiX.tiff *N* = total number of patches in class

The main folder describes the type, acquisition method, and sample information: ‘MultiRes’, indicates that the dataset contains images at acquired at multiple resolutions;, labelled as ‘ConfocFluo’ refers to the acquisition method: confocal fluorescence microscopy; ‘HumanDentin’ concerns the nature of the sample. The data repository was set up in this manner allowing for new datasets to be added as new acquisitions are taken in the future.

**Fig. 1:**
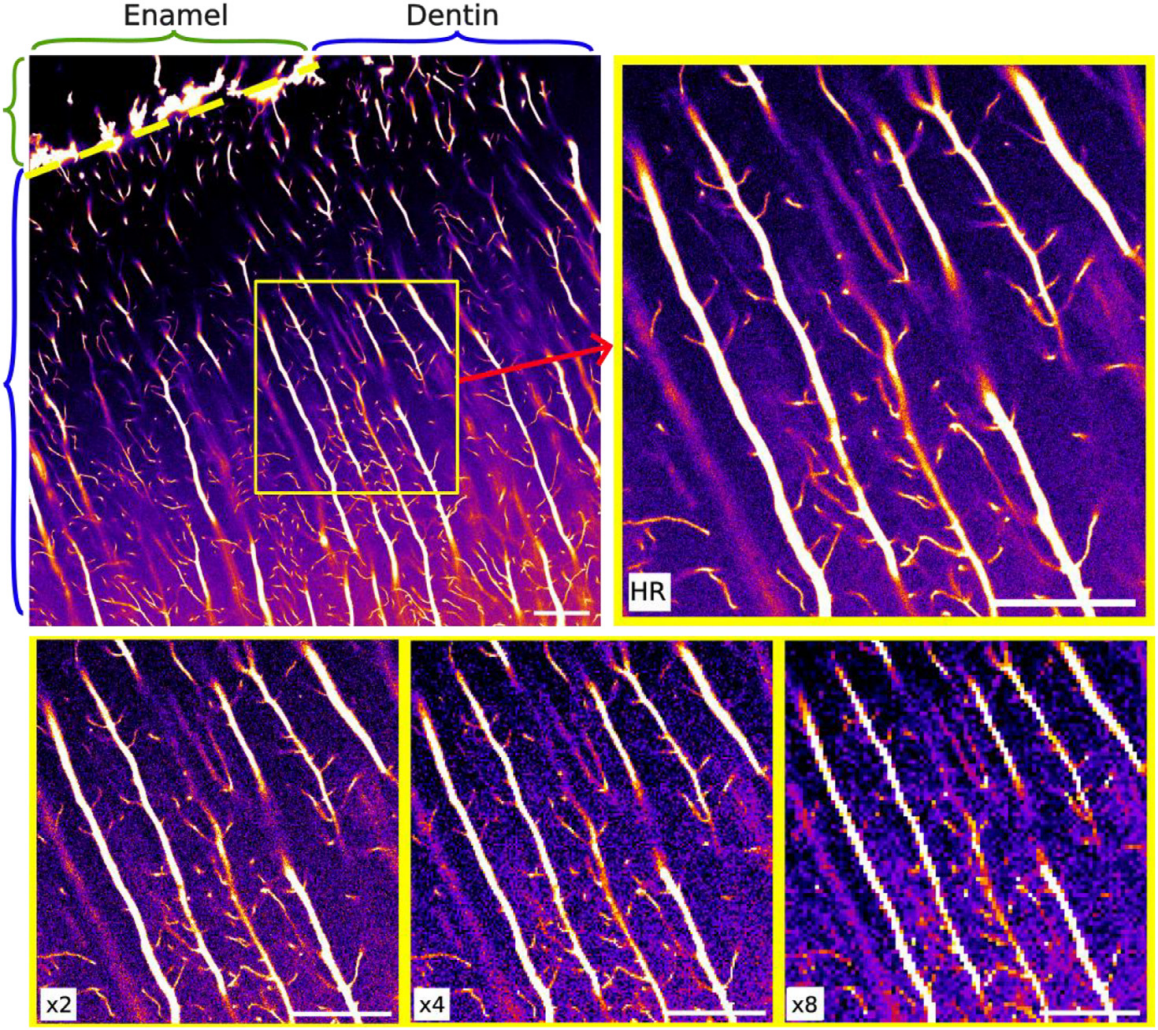
Full FOV for one high-resolution image with smaller region of interest for high-resolution and subsequent low-resolution images to better visualize tubules and branches. Decreasing resolutions show obvious degradation in quality and loss of information. Scale bar: 10 µm.

**Fig. 2:**
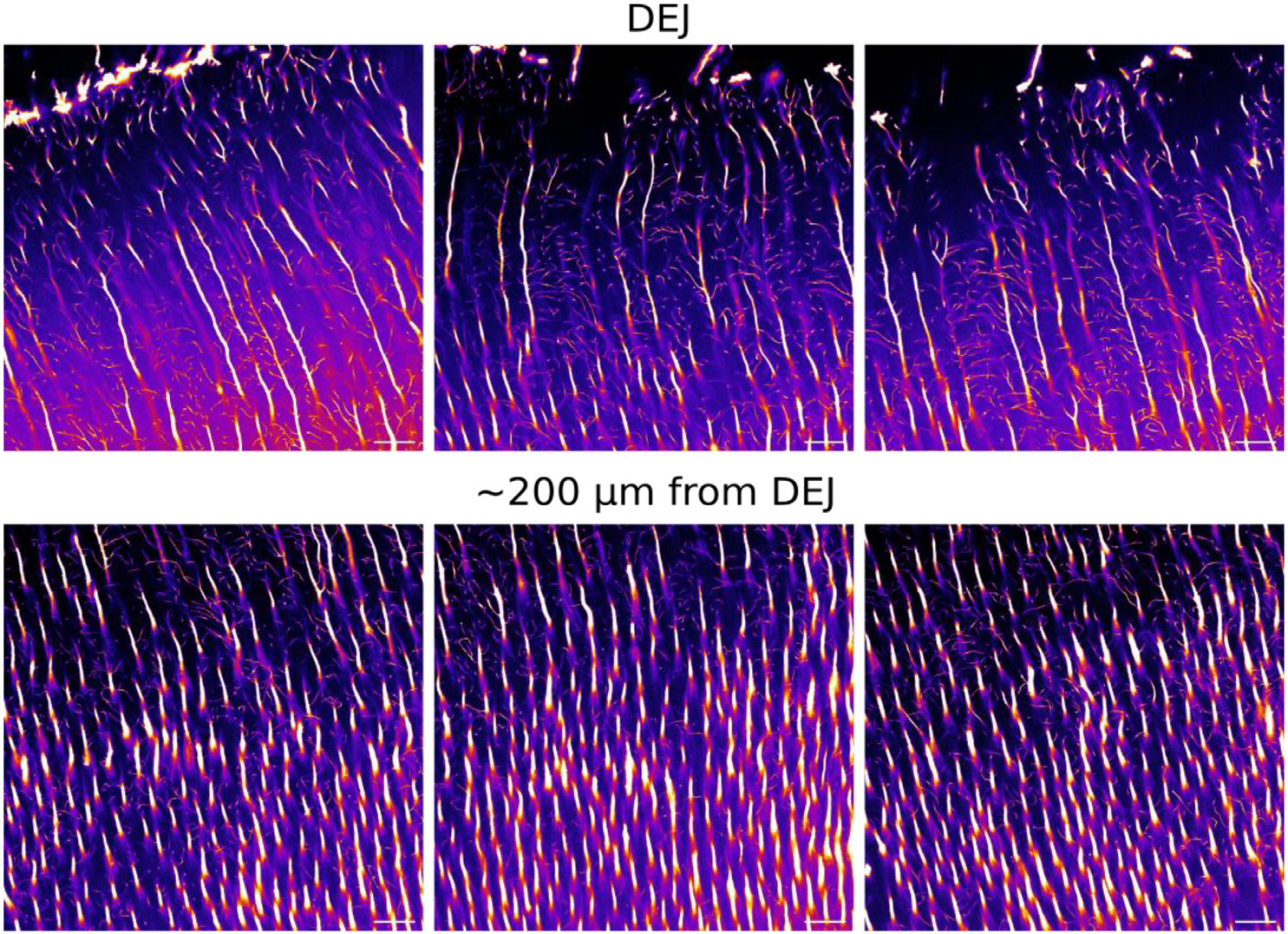
Image from each region acquired with their location relative the DEJ. Scale = 20 µm*.*

The first subfolder “RawImages”, contains the raw confocal laser scanning fluorescence microscopy images in 8-bit greyscale. The folder is subdivided by sample name, allowing for new samples to be added later, then by region, each region being subdivided by resolution. An example of all acquired resolutions for one region is shown in Fig. 1. A stack of 2D images was acquired at 2048×2048 pixels (100×100 nm^2^ pixel size) for high-resolution, and 1024×1024 pixels (200×200 nm^2^ pixel size), 512×512 pixels (400×400 nm^2^ pixel size), and 256×256 pixels (800×800 nm^2^ pixel size) for the subsequent low resolutions. The resulting field of view (FOV) for each image is 200×200 µm^2^. In total, 18 images were acquired for each FOV to create a 3D stack, with 0.3 µm step size in z resulting in a depth of 5.1 µm. Six acquisitions were taken near the dentin-enamel junction (DEJ), which is a functionally important region of the tooth. An image from each region is shown in Fig. 2 to illustrate structural differences. Files are named based on the sample, region of acquisition, and the z-slice of the image, where *z* = 0 is the first slice. For example, the image acquired in sample 1, region 1 at the first position of the z-stack would be named ‘s01_roi1_z0000.tif’.

The second subfolder “RegisteredImages” contains the low-resolution images, that have been pixel-to-pixel registered to the high-resolution images acquired in the same FOV. Low resolution images were resampled using nearest neighbour interpolation prior to registration to match the high-resolution image format (2048×2048 pixels) and were therefore saved with this sampling. These folders are sorted identically to “RawImages”, with data separated by sample, region, and then resolution.

**Fig. 3:**
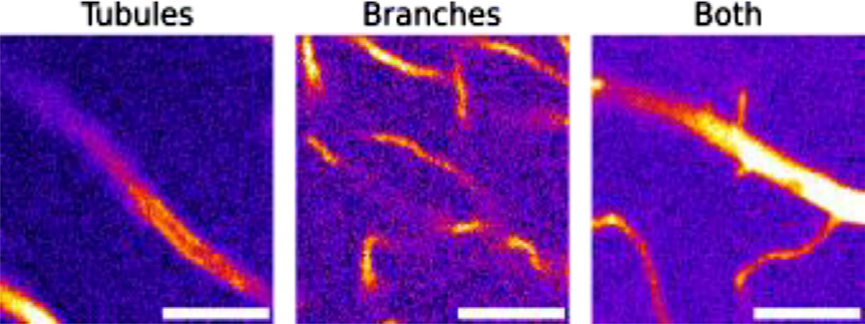
Example patch from each class. Scale = 5 µm.

Finally, the third subfolder, “ImagePatches128”, contains 12.8 × 12.8 µm^2^ (128×128 pixels) image patches separated into three different classes of porosity features: “Tubules” containing dentinal tubules, “Branches” containing dentinal branches and “Both” containing tubules and branches in the same patch. A folder ‘Classified’ contained folders for each class, with all image patches for the entire dataset saved in each respective class folder. An example of patches from each class is shown in Fig. 3. Images are labelled based on sample, region, and overall patch number, for example ‘img_00001_s01_roi1.tiff’ is patch number 00,001 from sample 1 and region 1. Including sample number allows the potential to add classified patches from different samples in the future to enrich the dataset.

Training models can therefore advantageously be achieved using data from the third subfolder, “ImagePatches128”. Paired models will require pairs of HR and corresponding LR patches at a desired resolution (x2, x4 or x8), which implies loading the same file number in the respective sub-folders. Alternatively, unpaired models should be trained using shuffled HR and LR files, i.e. with different file numbers.

## 4 Experimental Design, materials and Methods

### 4.1 Sample preparation

A healthy 28-year-old female human molar was extracted with informed oral consent in accordance with the ethical guidelines 153 laid down by French law (agreement IRB 00,006,477 and n° DC-2009–927, Cellule Bioéthique 154 DGRI/A5). The tooth was cleaned using an ultrasound bath and disinfected with Chloramine-T solution (0.5 % - 5 g/L). The tooth was fixed with 70 % ethanol for 48 h and embedded in athermal-curing epoxy resin (EPOFIX, Struers). A diamond saw (Struers E0D15 mounted on a Presi Mecatome T210) was used to slice the tooth to approximately 300 µm thick slices and one slice was hand-polished (Minitech 233, Presi) with 2000–4000 SiC abrasive paper (EU grade, Struers) and diamond polishing spray (DP-spray P, Struers) using aqueous lubricant oil (DP-lubricant red, Struers) from grain sizes of 3 µm down to 0.2 µm. The slice was polished until scratches from the diamond saw were removed and thickness was approximately 200 µm. The tooth slice was cleaned with water to remove any polishing residue and left to dry between two slide glasses for 48 h at room temperature. After drying, the sample was stained with 0.02 %wt rhodamine B (83,689–1 G, Sigma-Aldrich) and glycerol solution for 72 h, allowing for infiltration of the rhodamine B solution into the tubule and branch porosities. Finally, the sample was mounted between a slide glass and a cover slip, using double sided tape to secure the tooth slice to the slide glass. The rhodamine B with glycerol solution was added between the slide glass and cover slip as a mounting medium and the sample was sealed with cyanoacrylate glue. The mounted sample was left for 24 h to allow drying time for sealant before imaging. Double sided tape and cyanoacrylate glue were used to best try and reduce possible movement during imaging, which could introduce drift and difficulties when acquiring a paired dataset.

### 4.2 Image acquisition

Image acquisition was performed on a Leica TCS SP8 confocal microscope (Leica Microsystems CMS GmbH, Wetzlar, Germany), using the Leica Application Suite X (LASX) software to set parameters and control acquisition. The microscope and air conditioner in the experiment room were turned on the night prior to imaging and the sample was left in the room overnight to acclimate to the instrument and room temperature. This reduces the risk of drift caused by temperature shifts. A 40x oil objective with 1.3NA was chosen and the sample was mounted using a Leica immersion liquid with a refractive index of *n* = 1.52. A diode laser with a wavelength of λ = 561 nm was used to excite rhodamine B, and the emitted fluorescent light was detected using a Photomultiplier Tube (PMT) detector with a detecting range of [565 – 700 nm]. The theoretical point spread function of the microscope was measured to be 216 nm in x-y and 664 nm in z, calculated based on the objective specifications and emission wavelength using Abbe's diffraction limit as seen in Eq. (1) [8].

(1) $$d_{\left(x,y\right)}=\frac{\lambda }{2NA},d_{\left(z\right)}=\frac{2\lambda }{NA^{2}}.$$

High-resolution images were therefore acquired with a pixel size of 100nm^2^ pixel size in x-y and a step size of 0.3 µm in z. These values were chosen to slightly oversample Nyquist resolutions to ensure the best possible imaging. To create low-resolution images, the in-plane sampling factor was decreased by powers of 2. thus increasing the pixel size to under-sample the point-spread function (PSF) of the system. Line averaging was used for the high-resolution acquisition to optimize the signal/background ratio but was removed for low-resolution images to increase scan speed. Full scanning parameters for each resolution are outlined in Table 2. To improve image matching conditions between high- and low-resolution, the objective was never changed to maximize mechanical stability of the microscope.

**Table 2:** Experimental imaging parameters for each image acquisition at different resolutions.

Image Label	High Resolution	x2	x4	x8
Image Sampling (pixels)	2048×2048	1024×1024	512×512	256×256
Pixel Size (nm^2^)	100×100	200×200	400×400	800×800
Z-step size (µm)	0.3	0.3	0.3	0.3
Line Averaging	3	1	1	1
Scan Time (s)	264	46	23	13
Ratio of Pixels : PSF	2:1	1:1	0.5:1	0.25:1

Live sequence imaging was used to acquire images of all resolutions for each region with the lowest risk of mechanical or thermal drift with time. Images were acquired from lowest to highest resolution, to decrease time between different scans of the same plane. 200×200×5.1 µm^3^ stacks were acquired for each region and all resolutions were acquired for one region before manually moving to another. For simplicity, low-resolution stacks will be further referenced based on pixel size increase from high resolution image sampling (x2, x4, x8) as labelled in Table 2.

### 4.3 Image registration

While all efforts were made to acquire a completely paired dataset, some drift is inevitable during acquisition at the 100 nm precision level. Image registration was required to register the images on a pixel-to-pixel basis. The high-resolution stacks were used as the reference image. All low-resolution images were resampled using nearest neighbour interpolation to 2048×2048 pixels format to match that of the high-resolution dataset. Images were loaded into python and cropped into small 200×200 pixels regions of interest (ROI), selected manually by choosing a region that contained a structure that could be used to easily register the data. The ANTSPy library was used to calculate mutual information between the small ROIs (https://github.com/ANTsX/ANTsPy). The rigid transformation matrix found for the ROI was then applied to the entire stack to register each image. Since the depth acquired was limited to 5.1 µm, no Z-registration was required.

### 4.4 Balancing data

In the realm of deep learning model training, the sheer size of raw images necessitates the use of image patches as inputs due to memory constraints. Our approach to this involved a meticulous selection strategy: patches were categorized into three distinct classes based on their porosity features: “Tubules” (containing only dentinal tubules), “Branches” (containing only dentinal branches) and “Both” (containing tubules and branches in the same patch).

### 4.5 Mitigating bias through balanced representation

It’s generally understood that model performance is optimized when all classes are equally represented in the training dataset. This equal distribution is crucial for reducing bias, preventing the model from learning certain features better than others simply due to an overabundance of data or those features. Furthermore, it’s vital to ensure robust representation of small porosity features, as these small branches play a critical role in the connectivity of the porosity network.

To accommodate this, we select patch size of 12.8 × 12.8 µm^2^ (128×128 pixels). This dimension was chosen to ensure that patches could encapsulate both branches and tubules with the potential for up to two tubules within a single patch.

### 4.6 A pipeline for dataset balancing

**Fig. 4:**
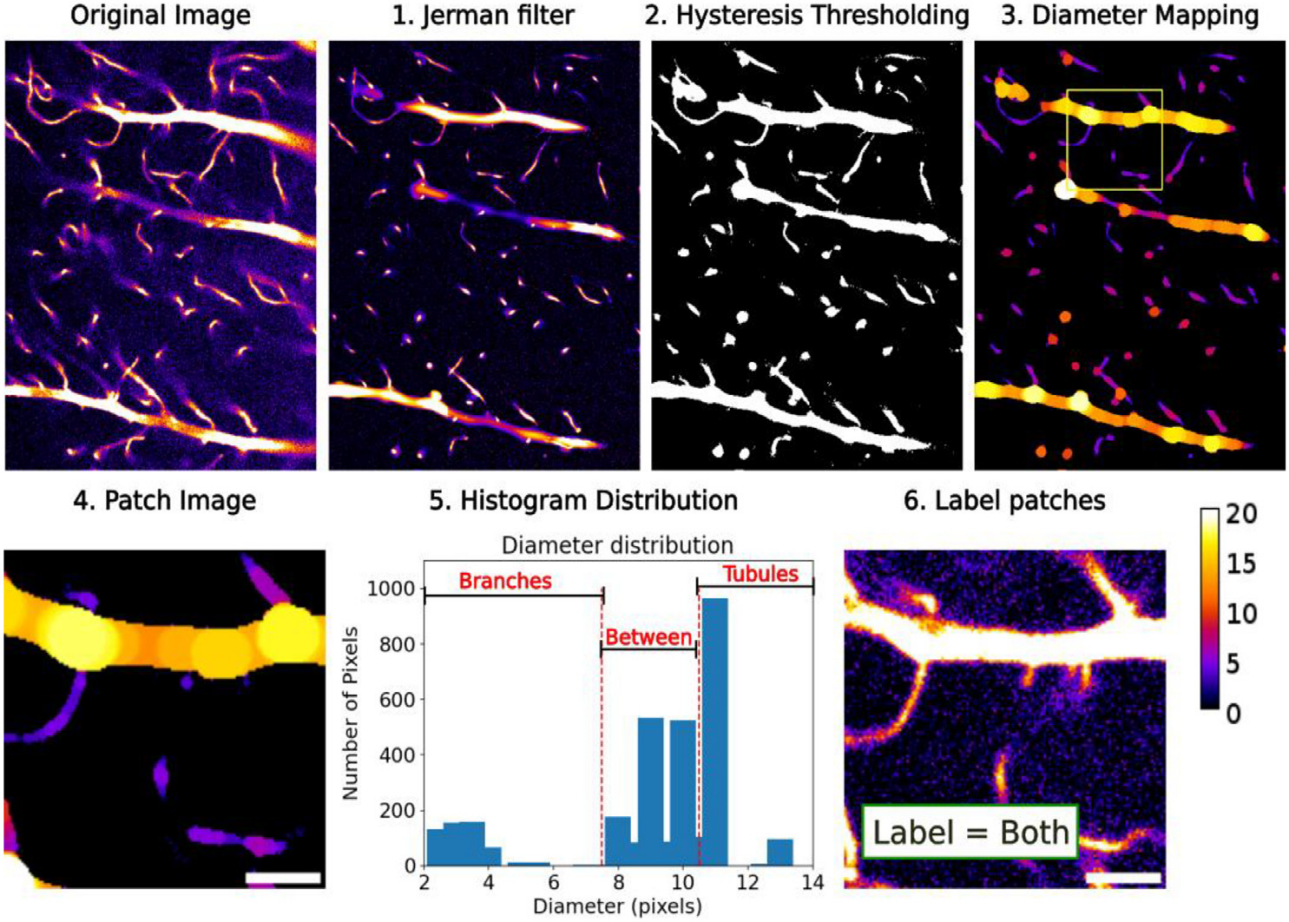
Illustration of the pipeline developed to label image patches and balance datasets. A crop of the original image followed by 1: Example of Jerman filtered data showing enhancement of tubular structures. 2: Example of binarized image calculated using hysteresis thresholding. 3: Example of diameter map where pixel value shows diameter of structure. 4: One example patch containing a tubule and branches. 5: Diameter histogram distribution for image patch. 6: Patch from the original image with class label found from the histogram distribution. Scale bar = 3µm.

To achieve a balanced dataset, we employed a proprietary a pipeline developed by our group. This pipeline systematically labels patches based on a histogram distribution of the structural diameters within each patch (as detailed in Fig. 4). It’s important to note that all image processing operations were initially applied to full, raw images. Patching was then performed as a final step, just prior to the histogram calculation that determined the content classification of each patch based on its diameter distributions.

First, Jerman’s implementation of a multiscale Vesselness enhancement filter [9,10] was applied to full image stacks to enhance tubular structures in the images using the pyvesselns library [11].Tubular structures are identified based on the eigenvalues of the hessian matrix. For an ideal tubular structure, one eigenvalue should be very small compared to the two others, as a result of the relatively constant intensity along the vessel. The other two should be approximately equal in case of a circular cross-section, but much larger than the first, reflecting a large change in intensity across tubules [9,10]. Scales were set as a range between 2 - 24 pixels with a step size of 0.5, which corresponds to 0.2–2.4 µm selected based on the smallest and largest possible FWHM across branches and tubules in the images. The filter sensitivity, τ, was set to 0.5. The filtered images have normalized pixel intensity. A hysteresis thresholding was applied to binarize the images, with upper and lower thresholds set at 0.15 and 0.4 respectively, based on the contrast of our images. Finally, a maximum fitting sphere algorithm was used to calculate the thickness of each structure using a modified version of the porespy library [12] . For each voxel in the structure, the radius of the largest sphere that fits in the foreground and engulfs the structure is taken as the diameter for that voxel. Each pixel is labelled accordingly, and final images represent a ‘diameter map’ of the original images.

Original high resolution, low resolution and high-resolution diameter map images were then split into 12.8 × 12.8 µm^2^ image patches, using 50 % overlap between patches to increase data size and reduce the number of lost connections occurring at patch edges. The histogram distribution for each diameter map image patch was calculated with a binning of 0.5 up to a maximum value of 40 to obtain the distribution of pixels for each diameter. Values below 2.5 pixels diameter were identified as noise and removed. Two thresholds were then determined: the first was set to 8, so any diameter values below were classified as branches; the second was set as 10, so any values above were classified as tubules. These values correspond to standard branch and tubule diameter, where normal range is from 300 to 700 nm for branches and 1 to 3 µm for tubules [2]. Pixels between these thresholds were classified as ‘between values’ and were disregarded in the overall classification to try and limit the misclassification of larger branches or small ends of tubules that can be close in diameter to their counterparts. Image patches were then labelled based on the percentage of pixels on either side of the histogram thresholds. A higher percentage was chosen for tubules compared to branches since tubules are larger and therefore contain more pixels. Furthermore, it was more important to ensure branches were well represented and therefore the branch percentage was set quite low to ensure even the smallest structures were counted and kept. The criteria for labelling patches are as follows:

> 0.5 % branches and > 5 % tubules: BOTH

> 0.5 % branches and < 5 % tubules: BRANCH

> 5 % tubules and < 0.5 % branches: TUBULE

Based on the labelling of the diameter patches, the corresponding original high- and low- resolution patches were also labelled. A total of 32,010 ‘Branch’ patches, 5201 ‘Tubule’ patches and 53,253 ‘Both’ patches were saved. Class labelling was fully done using thresholding based on branch and tubule size. Although this was the best approach beside manual labelling, the results are not completely perfect. There are cases where ‘Tubule’ patches contain some branches and where ‘Branch’ classes contain some small tubules. This was deemed acceptable since reasoning behind balancing classes was to ensure branches were well represented in the dataset and the balancing of classes ensured there was at least even representation of branches compared to tubules, with likely more branches than tubules. Furthermore, the quality of balancing the dataset is limited by the performance of the diameter map pipeline, and cases where small or low intensity features are missed during filtering or segmentation will therefore not be taken into account during patch labelling.

## Limitations

Understanding the inherent limitations in acquiring paired pixel-level data is paramount, especially when the process is hampered by the inevitable mechanical and thermal drift of the microscope. Our strategy to mitigate this major pitfall involved drastically reducing the scan time.

### Specifics of super-resolution experimental acquisition

To overcome the complex challenge of acquiring registered images at different spatial resolution with sub-PSF sampling, the choice was made to use the same objective and modify the scanning sampling parameters. Additional tests might be required for super-resolution models trained with our dataset to validate the use of different microscope objectives.

### Trade-offs between imaging depth and data richness

To constrain acquisition time, we prioritized reduced imaging depths along the Z-axis. While this approach undoubtedly minimized drift effects, it also, by necessity, limited the volume of data collected. This constraint could potentially restrict the exploitation of the database for exhaustive three-dimensional analyses.

### A pioneering and extensible data set

Despite these considerations, the data set we've compiled represents, to date, the largest paired data set of dentinal porosity, enabling multi-scale resolution analysis. Its architecture, founded on a rigorous protocol, paves the way for its future collaborative enrichment, inviting any contributor eager to expand its scope.

### Sample diversity and model generalizability

Imaging a single tooth sample introduces a limitation regarding the intrinsic diversity of the data. This homogeneity could potentially pose a challenge for the generalizability of models developed from this set. Furthermore, the exclusive focus on regions near the dentin-enamel junction (DEJ) restricts the representativeness of other dentin areas, which could prove crucial for future analyses and for increasing generalizability.

Consequently, our database should be viewed as an initial incentive for describing dentin porosity, whose expansion is desirable through increased collaborative efforts from the scientific community. Our team, for its part, intends to continue enriching this set, by incorporating images from diverse samples and regions.

### Data balancing precision and biological perspective

From a biological standpoint, the precise calibration of porosity features presents a significant challenge. Indeed, the morphology of the tubules change, becoming narrower and closer in diameter to branches near the DEJ. Since the balancing dataset protocol was performed using dimensional thresholds, there could be some slight misclassification. Our initial tests with super-resolution models nevertheless suggest considerable improvements from an unbalanced case scenario. Ultimately, since we also provide the raw data, users may also contribute by improving the classification depending on their needs.

## Ethics Statement

The authors declare that the tooth sample used in this study was obtained from the Faculty of Dental Surgery of Montrouge, France, following dental surgery performed at the Hôpital Henri Mondor, with verbal informed consent of the donor in accordance with the ethical guidelines laid down by French law (agreement IRB 00,006,477 and n° DC-2009–927, Cellule Bioéthique DGRI/A5). The verbal consent of the patient to the dentist was witnessed by his assistant.

## CRediT Author Statement

**Lauren Anderson:** Conceptualization, Data curation, Formal analysis, Investigation, Software, Visualization, Writing – original draft; **Kathryn Grandfield:** Conceptualization, Funding acquisition, Supervision, Writing - Review & Editing; **David Rousseau:** Conceptualization, Methodology, Supervision, Writing - Review & Editing; **Aurélien Gourrier:** Conceptualization, Funding acquisition, Project administration, Resources, Supervision, Writing - Review & Editing, Methodology.

## Data Availability

ZenodoMulti-resolution confocal fluorescence microscopy of human dentin porosity (Original data).
